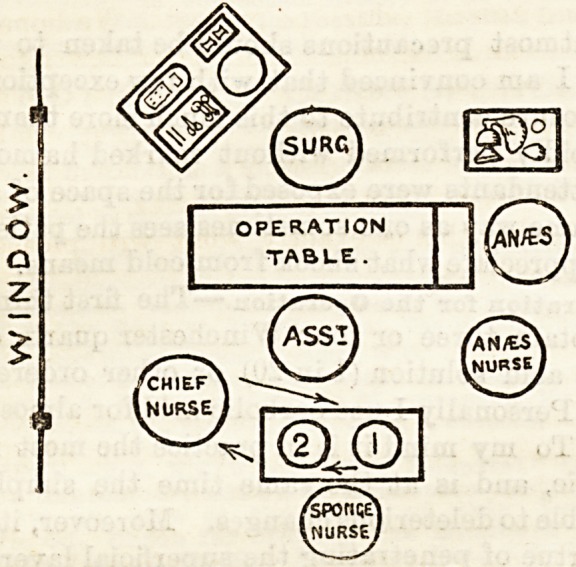# On the Preparation for and After-Treatment of Abdominal Operations

**Published:** 1898-12-24

**Authors:** Thomas Carwardine

**Affiliations:** Assistant Surgeon, Bristol Royal Infirmary.


### Hospital Clinics and Medical Progress.

### ON THE PREPARATION FOR AND AFTER-TREATMENT OF ABDOMINAL OPERATIONS.

By Thomas Carwardine, M.S.Lond., F.R.C.S.Eng., Assistant Surgeon, Bristol Royal Infirmary.

Although few are called upon to become abdominal operators, many are required to prepare the patient for the surgeon's visit and to carry out the general treatment subsequently. No other branch of surgery is so dependent upon faithfulness in little things on the part of the attendants. No make-shift in any detail is permissible, and thoroughness and perfection must be the constant aim. At least two nurses are required at first, one for night and one for day, and adequate outdoor recreation must be insisted on for each. The assistant should, as a rule, be accustomed to abdominal operations, and, if possible, to the individualities of the operator. In any case, he must be relied upon to keep his nails short, and thoroughly cleanse and disinfect his hands and forearms. He also, if possible, should be the assistant who may be called upon to act in subsequent emergency.

**The Operation Room** should be in a good light with cheerful prospect, preferably with a south aspect. It is convenient to have it near a bath-room, but it should be as far removed as possible from any water-closet. The carpet should be taken up and all furniture and upholstery not absolutely required must be removed. They are often heirlooms which have seen many a fever and perhaps not a few deaths. Then the room should be thoroughly disinfected, and whatever furniture remains cleansed and treated with a reliable antiseptic. The bed should be narrow, preferably with a horse-hair mattress, and one which will not "sag" in the middle.

**Preparation of the Patient.**—The patient should be kept in bed for a few days prior to operation upon a light diet. A purgative is given at least twenty-four hours before the operation, followed by a simple enema very early on the morning of operation.

The patient is thoroughly prepared the day before operation, first having a bath early morning, thoroughly cleansing the genitals and umbilicus, and getting into a clean, warmed bed to avoid any chill. The pubes should be shaved, and the whole abdomen thoroughly cleaned with a nail-brush and soap and water, then with clear turpentine, next with ether which dissolves the superfluous fat, and, lastly, with 1 in 40 carbolic. With pure hands an antiseptic compress is then applied, comprising at least two folds of lint wrung out of 1 in 60 carbolic, covered with jaconet and bandage, and renewed night and morning. This strength increases by evaporation, and stronger solutions are apt to excoriate the skin.

For the operation the patient should be clad in clean, well-aired flannel, comprising a vest, flannel jacket, drawers, and stockings. A large many-tailed bandage for the whole body and legs, made by cutting the sides of a blanket transversely, is a boon.

If the surgeon has ordered it, a boiled catheter (glass or soft rubber) is passed shortly before the surgeon's arrival; otherwise the patient may be allowed to pass urine naturally. The passage of the catheter ensures emptiness of the bladder, but unless everything it touches be made absolutely pure it is apt to set up cystitis.

The utmost precautions should be taken to prevent shock. I am convinced that, with few exceptions, cold and exposure contribute to this much more than operation rapidly performed without marked hæmorrhage. If the attendants were exposed for the space of an hour in the same way as one sometimes sees the patient they would appreciate what shock from cold means.

**Preparation for the Operation.**—The first thing to do is to obtain three or four Winchester quarts of pure carbolic acid solution (1 in 20) or other ordered antiseptic. Personally I use carbolic acid for almost everything. To my mind it is in practice the most reliable antiseptic, and is at the same time the simplest and least liable to deleterious changes. Moreover, it has the great virtue of penetrating the superficial layers of the skin.

In a private house the operation-table is usually prepared from two dressing-tables, one placed across the head of the other, and covered by a warm blanket, macintosh, and clean unbleached sheet. It may be kept warmed for the patient by means of indiarubber hot-water bottles. A large number of clean towels must be at hand, and several clean, recently-boiled basins and dishes of various sizes and shapes. Plenty of hot water should be ready in perfectly clean vessels, and a large saucepan or, preferably, a fish-kettle should be at hand with boiling water in case the surgeon should require the same for sterilisation purposes. A couple of perfectly new nail-brushes should be kept in 1 in 20 carbolic ready for use.

The prepared sponges and towels sterilised by boiling should be placed in to 1 in 20 carbolic some time before the operation, to be diluted to 1 in 40 with hot water just before use. The sponges and forceps must be reliably counted. A small blanket is required to go over the patient's chest, and a large one for the legs. The patient's legs should be fixed by a piece of strong webbing or bandage tied in a bow just above the knees, encircling the thighs and table. Macintoshes of jaconet are placed over the blankets. The bandages may be cut and removed with the jaconet by the general nurse, for the outer dressings are septic. Then the assistant replaces the antiseptic compress with a wet carbolised sponge cloth or piece of lint. Next the sterilised and carbolised towels are placed over the macintoshes, and last of all the assistant removes the temporary wet compress. The surgeon helps himself to his instruments, and the head nurse and sponge nurse keep themselves surgically pure, the anæsthetic nurse being available for other duties. No nurse should need to leave the room, unless perchance at the request of the surgeon. The head nurse hands (but does not handle) the sponges on a sterilised receiver, or in a bowl of antiseptic lotion, and receives the soiled sponges on another receiver. She is at hand also to thread needles, if required. The sponge nurse rinses the sponges in boiled water, and then in pure carbolic 1 in 40, placing them on the receiver of the head nurse as required. The most scrupulous care is necessary to ensure the purity of sponges. In many cases one nurse is sufficient for the sponges, the assistant taking the sponges as he requires them from a bowl of antiseptic. Every hand which touches a sponge is a source of danger.

All the operation attendants must thoroughly disinfect their hands. First the nails should be cut short, then the forearms and hands thoroughly scrubbed for three or four minutes with a new nail-brush, and soaked for two to three minutes in a reliable antiseptic. Personally, I prefer 1 in 40 carbolic; the fact that it makes the hands rough is part proof of its penetrating qualities.

(*To be continued.*)

## Figures and Tables

**Figure f1:**